# Allele-specific marker-based assessment revealed that the rice blast resistance genes *Pi2* and *Pi9* have not been widely deployed in Chinese *indica* rice cultivars

**DOI:** 10.1186/s12284-016-0091-8

**Published:** 2016-05-04

**Authors:** Dagang Tian, Zaijie Chen, Ziqiang Chen, Yuanchang Zhou, Zonghua Wang, Feng Wang, Songbiao Chen

**Affiliations:** College of Crop Science, Fujian Agricultural and Forestry University, Fuzhou, 350002 China; Biotechnology Research Institute, Fujian Academy of Agricultural Sciences, Fuzhou, 350003 China; Fujian-Taiwan Joint Center for Ecological Control of Crop Pests, Fujian Agriculture and Forestry University, Fuzhou, 350002 China

**Keywords:** Rice, Blast disease, Resistance gene, Molecular marker, *Pi2*, *Pi9*

## Abstract

**Background:**

The most sustainable approach to control rice blast disease is to develop durably resistant cultivars. In molecular breeding for rice blast resistance, markers developed based on polymorphisms between functional and non-functional alleles of resistance genes, can provide precise and accurate selection of resistant genotypes without the need for difficult, laborious and time-consuming phenotyping. The *Pi2* and *Pi9* genes confer broad-spectrum resistance against diverse blast isolates. Development of allele-specific markers for *Pi2* and *Pi9* would facilitate breeding of blast resistant rice by using the two blast resistance genes.

**Result:**

In this work, we developed two new markers, named Pi9-Pro and Pi2-LRR respectively, targeting the unique polymorphisms of the resistant and susceptible alleles of *Pi2* and of *Pi9*. The InDel marker Pi9-Pro differentiates three different genotypes corresponding to the *Pi2/Piz-t*, *Pi9* and non-*Pi2/Piz-t/Pi9* alleles, and the CAPS marker Pi2-LRR differentiates the *Pi2* allele from the non-*Pi2* allele. Based on the two newly developed markers and two available markers Pi2SNP and Pi9SNP, the presence of *Pi2* and *Pi9* was assessed in a set of 434 rice accessions consisting of 377 Chinese *indica* cultivars/breeding materials and 57 Chinese *japonica* cultivars/breeding materials. Of the 434 accessions tested, while one *indica* restorer line Huazhan was identified harboring the *Pi2* resistance allele, no other rice line was identified harboring the *Pi2* or *Pi9* resistance alleles.

**Conclusions:**

Allele-specific marker-based assessment revealed that *Pi2* and *Pi9* have not been widely incorporated into diverse Chinese *indica* rice cultivars. Thus, the two blast resistance genes can be new gene sources for developing blast resistant rice, especially *indica* rice, in China. The two newly developed markers should be highly useful for using *Pi2* and *Pi9* in marker-assisted selection (MAS) breeding programs.

**Electronic supplementary material:**

The online version of this article (doi:10.1186/s12284-016-0091-8) contains supplementary material, which is available to authorized users.

## Background

Rice blast, caused by the ascomycete fungus *Magnaporthe oryzae*, is the most destructive disease that affects rice production worldwide (Dean et al. 2005; Ebbole 2007). Deployment of resistant cultivars has been proved to be the most effective and environmentally-friendly way to control rice blast disease (Zeigler et al. 1994). Numerous resistance (*R*) genes conferring resistance to *M. oryzae* have been identified in diverse rice germplasm, and at least 23 blast *R*-genes have been cloned (Liu et al. 2014), providing a wealth of information and resources for improving blast resistance in rice.

Marker-assisted selection (MAS) has been employed to develop rice cultivars with resistance against blast disease. MAS allows rapid introgression of blast *R*-genes into susceptible varieties as well as pyramiding of multiple genes into a single line for more durable blast resistance. To date, a number of blast *R*-genes have been tagged by genetically linked DNA markers, such as SSR (simple sequence repeat) or other PCR-based markers. However, owing to genetic recombination, the genetically linked markers may give rise to false positives (Frisch et al. 1999). With the progress in cloning of blast *R*-genes and the availability of sequences of more functional *R*-genes, functional allele-specific markers have been developed for several blast *R*-genes, such as *Pi-km* (Costanzo and Jia 2010), *Pit* (Hayashi et al. 2010), *Pi54* (Ramkumar et al. 2011), *Pi2*/*Piz-t*/*Pi9* (Hua et al. 2015), and *Pita* (Jia et al. 2002; Ramkumar et al. 2015) by using the strategy of allele mining. These allele-specific markers provide more efficient selection of desired genotypes compared to DNA markers at nearby, but functionally irrelevant site (Andersen and Lubberstedt 2003).

The *Pi2/9* locus on the short arm of chromosome 6 contains at least nine identified blast *R*-genes, including *Pi26(t)* (Wu et al. 2005), *Pigm(t)* (Deng et al. 2006), *Piz(t)* (Fjellstrom et al. 2006), *Pi9* (Qu et al. 2006), *Pi2* (Zhou et al. 2006), *Piz-t* (Zhou et al. 2006), *Pi40(t)* (Jeung et al. 2007), *Pi2-2* (Jiang et al. 2012b), and *Pi50(t)* (Zhu et al. 2012). Previous studies showed that many *R*-genes from the *Pi2/9* locus confer broad-spectrum resistance to diverse *M. oryzae* isolates (Jiang et al. 2012b). The *Pi2/9* locus, therefore, has been extensively used in MAS breeding program for improving blast resistance in rice (Chen et al. 2004; Hayashi et al. 2006; Liu et al. 2008; Suh et al. 2009; Correa-Victoria and Martinez 2009; Wen and Gao 2011; Yin et al. 2011; Fu et al. 2012; Jiang et al. 2012a; Luo and Yin 2013; Jiang et al. 2015; Khanna et al. 2015). Three *R*-genes (*Pi2*, *Piz-t* and *Pi9*) from the *Pi2/9* locus have been cloned (Qu et al. 2006; Zhou et al. 2006). Sequence analyses revealed that *Pi2*, *Piz-t* and *Pi9* encode nucleotide binding site–leucine-rich repeat (NBS-LRR) type resistance proteins, and the genes are embedded in a cluster of tandemly repeated *NBS-LRR* genes (Qu et al. 2006; Zhou et al. 2006). DNA markers linked to the *Pi2* and *Pi9* genes have been used frequently for MAS breeding of rice in China (Chen et al. 2004; Liu et al. 2008; Wen and Gao 2011; Yin et al. 2011; Fu et al. 2012; Jiang et al. 2012a; Jiang et al. 2015). Although genetically linked markers were suitable for screening, DNA markers targeting the polymorphisms that define the functional and non-functional alleles of *Pi2* and of *Pi9* would greatly enhance the efficiency and accuracy of genotype selection in MAS breeding programs. In the present study, we present the development of two allele-specific markers for *Pi2* and *Pi9*. The markers were further used to assess the presence of the two blast *R*-genes in 434 rice accessions, consisting of 377 Chinese *indica* cultivars/breeding materials and 57 Chinese *japonica* cultivars/breeding materials. Among the 434 accessions, only the *indica* restorer line Huazhan was identified harboring the *Pi2* resistance allele, indicating that the two blast *R*-genes have not been widely incorporated into diverse Chinese *indica* rice cultivars. This suggests that *Pi2* and *Pi9* could be important new sources for developing blast resistant rice in China, especially in *indica* rice. The two newly developed markers should facilitate rapid incorporations of these resistance genes into rice through MAS breeding programs.

## Results

### Development of a marker targeting a conserved InDel in the promoter region of *Pi9*

**Fig. 1:**
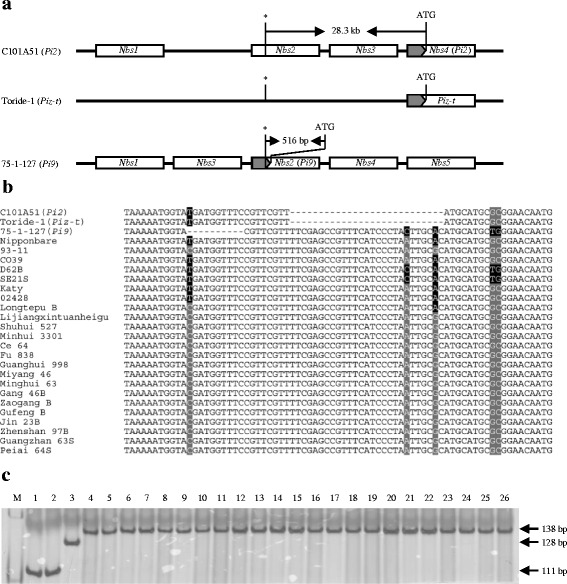
An InDel marker targeting the promoter region of *Pi9*. **a** Physical location of the identified InDel region in the *Pi2/9* locus. **b** Sequence alignment showing the 27-bp InDel between the *Pi2/Piz-t* and non-*Pi2/Piz-t* alleles and the 10-bp InDel between the *Pi9* and non-*Pi9* alleles in diverse cultivars. **c** PCR amplification patterns of the InDel marker Pi9-Pro that differentiates the *Pi2/Piz-t*, *Pi9* and non-*Pi2/Piz-t/Pi9* alleles. M: DNA ladder; 1: C101A51 (*Pi2* donor line); 2: Toride-1 (*Piz-t* donor line); 3: 75-1-127 (*Pi9* donor line); 4: Nipponbare; 5: 93–11; 6: CO39; 7: D62B; 8: SE21S; 9: Katy; 10: 02428; 11: Longtepu B; 12: Lijiangxintuanheigu; 13: Shuhui 527; 14: Minhui 3301; 15: Ce 64; 16: Fu 838; 17: Guanghui 998; 18: Miyang 46; 19: Minghui63; 20: Gang 46B; 21: Zaogang B; 22: Gufeng B; 23: Jin 23B; 24: Zhenshan 97B; 25: Guangzhan 63S; 26: Peiai 64S. Asterisk indicates the InDel region; Grey arrows indicate the promoter regions of *Pi2*, *Piz-t* or *Pi9*

Genomic DNA sequences corresponding to the *Pi2/9* locus in cvs. C101A51 (*Pi2*-carrying isogenic line), 75-1-127 (*Pi9*-carrying line), and Nipponbare were retrieved from Genbank and were used for comparative analysis. Because InDel (insertion-deletion) markers are more convenient for use in most regular laboratories compared with SNP (single nucleotide polymorphism) markers, we focused firstly on InDel polymorphisms among the sequences and found that the region corresponding to the promoter of *Pi9* and its alleles contains InDel polymorphisms with potential as molecular markers (Fig. 1a). To further verify the identified InDel polymorphisms, PCR fragments corresponding to the promoter region of *Pi9* and its alleles were amplified from C101A51, Toride-1 (*Piz-t*-carrying line), 75-1-127, Nipponbare, and 22 other cultivars (Fig. 1b) and were sequenced. Through multiple sequence alignment, a 10-bp InDel variation positioned at −516/-517 bp in the promoter region of *Pi9* in 75-1-127 (relative to the ATG start codon of *Pi9*) was identified that was not present in any of the other 25 cultivars (Fig. 1a-b). Interestingly, a 27-bp InDel close to the 10-bp InDel was also identified in sequences from C101A51 and Toride-1 that was not found in the other 24 cultivars (Fig. 1b). This 27-bp InDel is located about 28.3 kb upstream of the coding region of *Pi2/Piz-t* (Fig. 1a). Based on the sequences alignment result, a pair of specific primers, 9-Pro-F/9-Pro-R (Additional file 1: Table S1), targeted to the region containing the identified InDels was designed to develop molecular marker. Using the primers 9-Pro-F/9-Pro-R, PCR analysis detected an expected 111-bp fragment in both C101A51 and Toride-1, and an expected 128-bp fragment in 75-1-127 (Fig. 1c). In contrast, PCR analysis detected a 138-bp fragment in all other cultivars (Fig. 1c). Therefore, the InDel marker (named Pi9-Pro) can differentiate three different genotypes corresponding to the *Pi2/Piz-t*, *Pi9* and non-*Pi2/Piz-t/Pi9* alleles.

To validate the linkage of the Pi9-Pro marker with the *Pi2*- and *Pi9*-resistance phenotype, two F_2_ populations from the crosses of C101A51 × CO39 and 75-1-127 × CO39 respectively, were inoculated with the *M. oryzae* isolate PO6-6 which was incompatible to both *Pi2* and *Pi9*. In the F_2_ segregating population of C101A51 × CO39, PCR analysis based on primers 9-Pro-F/9-Pro-R detected a 111-bp fragment in either homozygous or heterozygous patterns in all 134 resistant plants. By contrast, PCR amplification yielded only a 138-bp fragment in all 40 susceptible plants. Similarly, the 128-bp genotype of the Pi9-Pro marker corresponding to *Pi9* allele was found to co-segregate with the blast-resistance phenotype in the F_2_ segregating population of 75-1-157 × CO39.

### Development of a CAPS marker targeting the LRR domain of *Pi2*

**Fig. 2:**
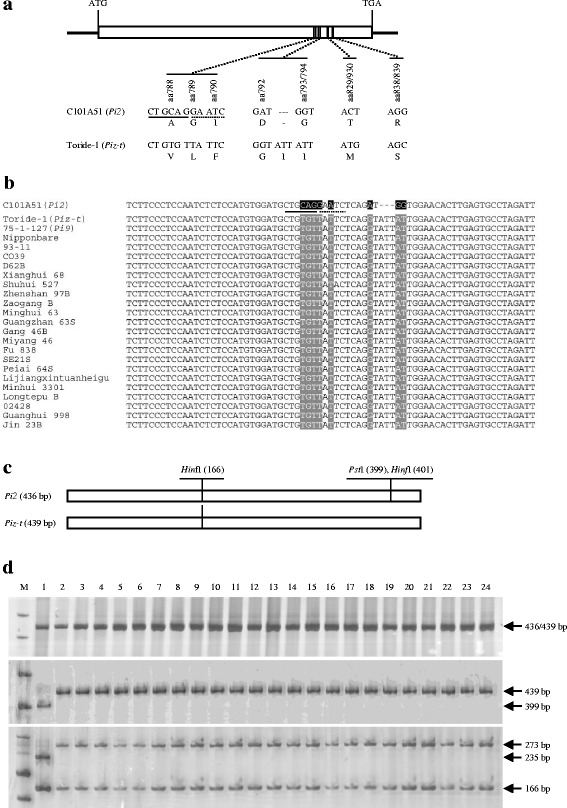
A CAPS marker targeting the LRR domain of *Pi2*. **a** DNA polymorphisms corresponding to the eight amino-acid differences in between Pi2 and Piz-t. **b** Sequence alignment showing that the polymorphic variations corresponding to the first six amino-acid differences in between Pi2 and Piz-t were conserved between the *Pi2* and non-*Pi2* alleles in diverse cultivars. **c** Schematic diagrams indicating sizes and recognition sites for *Hin*fI and *Pst*I in the PCR fragment of the *Pi2* and *Piz-t* alleles amplified with primers 2-LRR-F/2-LRR-R. **d** Electrophoresis profiles of the CAPS marker Pi2-LRR that differentiates the *Pi2* and non-*Pi2* alleles. Upper panel showing the PCR products without digestion with restriction enzymes, middle panel showing the PCR products digested with *Pst*I, and lower panel showing the PCR products digested with *Hin*fI. M: DNA ladder; 1: C101A51 (*Pi2* donor line); 2: Toride-1 (*Piz-t* donor line); 3: 75-1-127 (*Pi9* donor line); 4: Nipponbare; 5: 93–11; 6: CO39; 7: D62B; 8: Xianghui 68; 9: Shuhui 527; 10: Zhenshan 97B; 11: Zaogang B; 12: Minghui 63; 13: Guangzhan 63S; 14: Gang 46B; 15: Miyang 46; 16: Fu 838; 17: SE21S; 18: Peiai 64S; 19: Lijiangxintuanheigu; 20: Minhui 3301; 21: Longtepu B; 22: 02428; 23: Guanghui 998; 24: Jin 23B. *Pst*I recognition sequence (CTGCAG) is underlined; *Hin*fI recognition sequence (GAATC) is marked by dotted underling

The Pi9-Pro marker was unable to differentiate between the *Pi2* and *Piz-t* alleles. To further develop a marker that can be used to discriminate *Pi2* from *Piz-t* or from other non-*Pi2* alleles, we focused on the polymorphic sites within the coding regions of *Pi2* and *Piz-t*. As previously reported, Pi2 and Piz-t displayed eight amino-acid differences within three leucine-rich repeat (LRR) motifs (Fig. 2a) (Zhou et al. 2006). Alignment of DNA sequences of *Pi2* and its alleles from C101A51, Toride-1, 75-1-127, and Nipponbare revealed that the polymorphic variations corresponding to the first six amino-acid differences (AA788, AA789, AA790, AA792, AA---/793, and AA793/794; Fig. 2a) were conserved among different alleles (Fig. 2b). To further verify the polymorphisms for marker development, PCR fragments corresponding to the identified polymorphic region were amplified from C101A51, Toride-1, 75-1-127, Nipponbare and 20 other cultivars (Fig. 2b), and were sequenced. Multiple sequence alignment of the PCR products revealed that the polymorphic variations were conserved between *Pi2* and alleles of the other 23 tested cultivars (Fig. 2b). Sequence analysis also revealed that the variations defining the functional *Pi2* gene formed two recognition sites located closely together for restriction endonucleases *Pst*I and *Hin*fI, respectively (Fig. 2c).

Based on the sequence variations, a CAPS (cleaved amplified polymorphic sequence) marker (named Pi2-LRR) was designed for *Pi2*. Using a pair of specific primers, 2-LRR-F/2-LRR-R (Additional file 1: Table S1), PCR amplification yielded fragments of 436 bp, or 439 bp for *Pi2* and *Piz-t* alleles, respectively (Fig. 2c-d). Upon digestion with *Pst*I or *Hin*fI, the PCR products showed discernible patterns between the *Pi2* and *Piz-t* alleles (Fig. 2c-d). For example, following digestion with *Pst*I, the *Pi2*-PCR product was reduced to a 399 bp fragment, and the *Piz-t*-PCR product remained intact with its original size of 439 bp. Using the 2-LRR-F/2-LRR-R primers, PCR fragments were successfully amplified from genomic DNAs of C101A51, Toride-1, 75-1-127, Nipponbare and 20 other cultivars. Digestion analysis of the PCR products showed that the Pi2-LRR marker could differentiate the *Pi2* allele from the *non-Pi2* allele in diverse rice cultivars (Fig. 2d). The Pi2-LRR marker was also validated in a F_2_ segregating population derived from the cross of C101A51 × CO39, and the result showed that the genotype corresponding to *Pi2* allele for the Pi2-LRR marker co-segregated with the blast-resistance phenotype.

### Assessment of the *Pi2* and *Pi9* genes in diverse Chinese *indica* rice cultivars

The Pi9-Pro and Pi2-LRR markers were further validated for their specificity to *Pi2* or *Pi9* in monogenic lines harboring blast resistance genes in the *Pi2/9* locus, including IRBLz5-CA (*Piz-5* = *Pi2*), IRBL9-W (*Pi9*), IRBLzt-T (*Piz-t*) and IRBLz-Fu (*Piz*) (Tsunematsu et al. 2000). PCR analysis based on primers 9-Pro-F/9-Pro-R detected a *Pi9*-type genotype and a *Pi2*/*Piz-t*-type genotype in IRBL9-W and in IRBLz5-CA/IRBLzt-T (Table 2), respectively. As for the Pi2-LRR marker, PCR analysis detected a *Pi2*-type genotype only in IRBLz5-CA (Table 2).

**Fig. 3:**
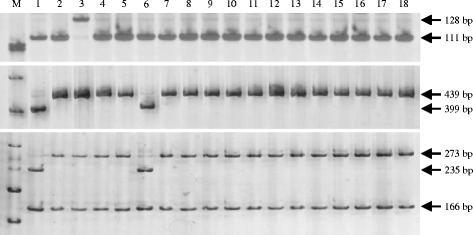
Pi9-Pro and Pi2-LRR analyses of 15 cultivars displaying a *Pi2/Piz-t*-type genotype for the Pi9-Pro marker. Upper panel showing the PCR amplification patterns of the InDel marker Pi9-Pro; Middle panel showing the patterns of Pi2-LRR products digested with *Pst*I; and lower panel showing the patterns of Pi2-LRR products digested with *Hin*fI. M: DNA ladder; 1: C101A51 (*Pi2* donor line); 2: Toride-1 (*Piz-t* donor line); 3: 75-1-127 (*Pi9* donor line); 4: Duohui 43; 5: Minghui 86; 6: Huazhan; 7: Minghui 1259; 8: Xianghui 68; 9: Fengxinzhan; 10: GIZA 176; 11: Jiabala; 12: Jiazao No.1; 13: Jiazao 935; 14: Haomake (K); 15: Lemont; 16: Shuiyuan 290; 17: Shuiyuan 377; 18: Zhonghua No.8

Besides the donor and monogenic lines, a set of 434 Chinese rice cultivars or breeding materials used in China were assessed for the presence of the *Pi2* and *Pi9* genes. The 434 rice accessions consisted of 91 *indica* restorer lines of hybrid rice, 61 *indica* maintainer/sterile lines of hybrid rice, 225 *indica* conventional cultivars/breeding materials, and 57 *japonica* conventional cultivars/breeding materials (Additional file 2: Table S2). When using the Pi9-Pro marker for screening, 15 out of 434 accessions were identified displaying a 111-bp PCR fragment (Table 1, Table 2, Fig. 3), suggesting that these 15 accessions might contain the *Pi2* or *Piz-t* resistance genes. In contrast, no accessions were detected harboring a 128-bp PCR fragment corresponding to the *Pi9* allele (Table 1), suggesting that the tested accessions did not have the *Pi9* resistance gene. When using the Pi2-LRR marker for screening, no tested accession except an *indica* restorer line Huazhan produced digestion patterns identical to that of the *Pi2* allele (Table 1, Table 2, Fig. 3). Notably, Huazhan was among the 15 accessions that displayed a 111-bp PCR fragment corresponding to the *Pi2*/*Piz-t* alleles as identified by the Pi9-Pro marker. Taken together, these results suggested that Huazhan might contain the *Pi2* resistance gene.

**Table 1:** Summary of genotyping results of the four molecular markers in 434 Chinese rice cultivars or breeding materials

Marker	Genotype (bp)	Corresponding donor line	No. of cultivars or breeding materials					Total
				Indica			Japonica	
				Restorer lines of hybrid rice	Maintainer or sterile lines of hybrid rice	Conventional cultivars or breeding materials	Conventional cultivars or breeding materials	
			Total	91	61	225	57	434
Pi9-Pro	138			86	61	220	52	419
	128	75-1-127 (*Pi9*)		0	0	0	0	0
	111	C101A51 (*Pi2*) Toride-1 (*Piz-t*)		5	0	5	5	15
Pi2-LRR	*Pst*I-439			90	61	225	57	433
	*Pst*I-399	C101A51 (*Pi2*)		1	0	0	0	1
	*Hin*fI-273			90	61	225	57	433
	*Hin*fI-235	C101A51 (*Pi2*)		1	0	0	0	1
Pi2SNP^a^	267			90	61	225	57	433
	235	C101A51 (*Pi2*)		1	0	0	0	1
Pi9SNP^b^	126			91	61	225	57	434
	108	75-1-127 (*Pi9*)		0	0	0	0	0

^a^ *Pi2*-type genotype for Pi2SNP is 235 bp

^b^ *Pi9*-type genotype for Pi9SNP is 108 bp

**Table 2:** List of the 15 cultivars displaying a *Pi2/Piz-t*-type genotype for the Pi9-Pro marker

Subspecies	Category	Cultivars	Molecular marker genotype (bp)					Disease reactions									
			Pi9-Pro	Pi2-LRR		Pi2SNP^a^	Pi9SNP^b^	KJ201	CHE86	CHNOS	RB16	RB22	HYG99	IR16	JL311	JL501	ZY838
				*Pst*I	*Hin*fI												
	Reference line	Nipponbare	138	439	273	267	126	S	S	S	S	S	S	S	S	S	S
	Donor lines	C101A51 (*Pi2*)	111	399	235	235	126	R	R	S	R	S	R	R	R	R	R
		Toride-1 (*Piz-t*)	111	439	273	267	126	R	R	R	S	S	S	S	S	S	S
		75-1-127 (*Pi9*)	128	439	273	267	108	R	R	R	R	R	R	R	R	R	R
	Monogenic lines	IRBLz5-CA (*Pi2*)	111	399	235	235	126	ND	ND	ND	ND	ND	ND	ND	ND	ND	ND
		IRBLzt-T (*Piz-t*)	111	439	273	267	126	ND	ND	ND	ND	ND	ND	ND	ND	ND	ND
		IRBL9-W (*Pi9*)	128	439	273	267	108	ND	ND	ND	ND	ND	ND	ND	ND	ND	ND
		IRBLz-FU (*Piz*)	138	439	273	267	126	ND	ND	ND	ND	ND	ND	ND	ND	ND	ND
Indica	Restorer lines of hybrid rice	Duohui 43	111	439	273	267	126	R	R	R	R	R	R	R	S	S	S
		Minghui 86	111	439	273	267	126	R	R	R	R	S	R	R	S	R	R
		Huazhan	111	399	235	235	126	R	R	R	R	R	R	R	R	R	R
		Minghui 1259	111	439	273	267	126	R	R	R	R	S	R	S	S	S	R
		Xianghui 68	111	439	273	267	126	R	R	R	S	S	S	R	S	S	S
	Conventional cultivars and breeding materials	Fengxinzhan	111	439	273	267	126	R	R	R	R	R	S	R	S	S	R
		GIZA 176	111	439	273	267	126	R	R	R	R	S	S	R	R	R	R
		Jiabala	111	439	273	267	126	R	R	R	R	R	S	S	R	R	R
		Jiazao No.1	111	439	273	267	126	R	R	R	R	R	R	R	S	R	R
		Jiazao 935	111	439	273	267	126	R	R	R	S	S	S	S	S	S	R
Japonica	Conventional cultivars and breeding materials	Haomake (K)	111	439	273	267	126	R	R	R	R	S	R	R	S	S	R
		Lemont	111	439	273	267	126	R	R	R	R	R	R	R	S	R	R
		Shuiyuan 290	111	439	273	267	126	R	R	R	R	S	R	R	R	S	R
		Shuiyuan 377	111	439	273	267	126	R	R	R	R	R	S	S	S	S	S
		Zhonghua No.8	111	439	273	267	126	R	R	R	R	R	S	S	S	R	S

*S* susceptible, *R* resistant, *ND* not determined

^a^ *Pi2*-type genotype for Pi2SNP is 235 bp

^b^ *Pi9*-type genotype for Pi9SNP is 108 bp

The 434 rice accessions were also screened with two CAPS/dCAPS (derived cleaved amplified polymorphic sequence) markers Pi2SNP and Pi9SNP which were developed based on SNPs within the coding region of *Pi2* and *Pi9*, respectively (Hua et al. 2015). The genotyping results of the Pi2SNP and Pi9SNP markers were consistent with that of the Pi2-LRR and Pi9-Pro markers. Pi2SNP analysis identified only one (cv. Huazhan) out of 434 accessions giving a restriction enzyme digestion pattern identical to that of the *Pi2* allele (Table 1, Table 2), and no accession was identified having a Pi9SNP genotype corresponding to the *Pi9* allele (Table 1).

### Disease reaction of cultivars possessing a *Pi2/Piz-t*-type genotype for the Pi9-Pro marker

Fifteen accessions were identified having a Pi9-Pro genotype identical to that of *Pi2/Piz-t* alleles (Table 2). Further genotyping of the 15 cultivars using Pi2-LRR and Pi2SNP markers strongly suggested that Huazhan harbors the *Pi2* resistance gene. To investigate whether the *Pi2/Piz-t-*type cultivars showed *Pi2*- or *Piz-t*-specific resistance, the 15 cultivars were inoculated with two blast isolates KJ201 and CHE86, which were incompatible to *Pi2*, *Piz-t* and *Pi9*; one isolate, CHNOS, which was compatible to *Pi2*, but incompatible to *Piz-t* and *Pi9* (Zhou et al. 2006); and seven isolates collected from Fujian province, China, which were compatible to *Piz-t*. As shown in Table 2, the *Pi2*-type cultivar Huazhan appeared resistant to isolates KJ201, CHE86 and CHNOS, consistent with the disease reactions in the *Pi2*-carrying isogenic line C101A51, except that C101A51 was susceptible to CHNOS. The other 14 *Piz-t-*type cultivars showed resistance to KJ201, CHE86 and CHNOS, consistent with the disease reactions in the *Piz-t*-carrying line Toride-1. As for inoculations with the seven Fujian isolates, the 15 cultivars showed differences in resistance spectrum compared with the *Pi2* or *Piz-t* donor lines, suggesting that Huazhan and the 14 *Piz-t*-type cultivars probably possess other blast *R*-genes conferring resistance to CHNOS or the Fujian isolates.

## Discussion

The use of resistant cultivars is an effective approach to control crop diseases. Over the past many decades, conventional breeding has had great success in improving rice resistance to blast disease. However, the traditional phenotype-based approach for breeding resistant cultivars is extremely laborious and time-consuming, and is often hampered by difficulty in screening the desired resistant progeny under field or limited inoculation conditions. In recent years, MAS breeding programs are being applied as an important supplement to the conventional phenotypic selection. Based on DNA molecular markers linked to resistance genes, MAS permits the direct selection of genes that control the disease resistance phenotype, providing an alternative to overcome the limitations of conventional breeding (Jena and Mackill 2008).

The efficiency of MAS mainly depends on the convenience of the use of DNA markers and the degree of linkage of the markers with the desired traits. Three major types of DNA markers, SSR, InDel and SNP markers have been frequently used in rice research and molecular breeding. Because of their co-dominance, capacity of multi-allelic detection and ease of use, SSR markers have been widely used for genotyping and MAS programs in rice (McCouch et al. 2002). For example, many blast *R*-genes were mapped based on SSR markers (Ashkani et al. 2016), and these markers linked to the blast *R*-genes have been thus applied in breeding of blast-resistant rice. However, due to the relatively low density of SSR markers, most of these SSR markers did not completely co-segregate with the mapped blast *R*-genes, which can lead to false positive selections. SNP and InDel polymorphisms are very abundant in the rice genome, providing potential targets for designing allele-specific markers directly targeting distinct blast-*R* gene alleles (Costanzo and Jia 2010; Hayashi et al. 2010; Ramkumar et al. 2011; Hua et al. 2015; Ramkumar et al. 2015). Because of their co-segregation with target blast *R*-genes (Ramkumar et al. 2011; Ramkumar et al. 2015), allele-specific markers are more effective than the random genomic SSR marker, allowing for selection of target blast *R*-genes with less labor and cost in MAS programs.

The *Pi2* and *Pi9* genes confer broad-spectrum resistance against diverse *M. oryzae* isolates (Liu et al. 2002; Qu et al. 2006; Zhou et al. 2006). Although several DNA markers have been developed for *Pi2* (Chen et al. 2004; Liu et al. 2008; Fu et al. 2012; Jiang et al. 2012a; Jiang et al. 2015; Khanna et al. 2015; Hua et al. 2015) and *Pi9* (Wen and Gao 2011; Yin et al. 2011; Luo and Yin 2013; Khanna et al. 2015; Hua et al. 2015), many of the markers were designed based on SSR polymorphisms or structural variants located some distance from the *Pi2* or *Pi9* genes. More recently, two CAPS/dCAPS markers targeting SNPs in the coding regions of *Pi2* and *Pi9* were reported (Hua et al. 2015). However, these two markers have not been validated for blast resistance. In the present study, we developed two new markers, Pi9-Pro and Pi2-LRR, targeting the unique polymorphisms in between the resistant and susceptible alleles of the two blast-R genes, respectively. The newly developed Pi9-Pro marker differentiates three different genotypes corresponding to the *Pi2/Piz-t*, *Pi9*, and non-*Pi2/Piz-t/Pi9* alleles. The Pi9-Pro marker was validated in F_2_ segregating populations, and the results confirmed that both the *Pi2-*type and *Pi9-*type genotypes for Pi9-Pro perfectly co-segregated with the blast resistance phenotype. The Pi9-Pro marker was developed based on InDel polymorphisms. Unlike CAPS/dCAPS markers (Hua et al. 2015) which require digestion of PCR amplicons for genotyping, the newly developed Pi9-Pro InDel marker eliminates this extra laborious step, and reduces potential sources of error (Liu et al. 2015). Thus, the Pi9-Pro marker is advantageous for MAS breeding programs targeting these resistance genes, especially for large-scale population screening. The Pi9-Pro marker, however, was unable to differentiate between the *Pi2* and *Piz-t* alleles. Pi2-LRR is a CAPS marker targeting the LRR motifs of the coding region of *Pi2*. The Pi2-LRR marker differentiates the *Pi2* allele from the *Piz-t* allele, and from other non-*Pi2* alleles. Pi2-LRR was also validated in a F_2_ segregating population, which showed that the *Pi2-*type genotype of the marker co-segregated with the blast resistance phenotype. Thus, when Pi9-Pro serves as a base marker for large-scale screening of *Pi2* in MAS breeding programs, Pi2-LRR can be used as a supplementary marker for confirmative screening, enabling more precise selection of *Pi2* in rice breeding populations.

To further assess the potential value of the *Pi2* and *Pi9* genes in breeding of blast resistant rice in China, a set of 434 Chinese rice cultivars or breeding materials, mainly consisted of *indica* cultivars, were screened with the Pi9-Pro and Pi2-LRR markers as well as the previously developed markers Pi2SNP and Pi9SNP (Hua et al. 2015). The screening revealed only one out of 434 rice accessions possessed the *Pi2* resistance allele, and no cultivar possessed the *Pi9* resistance allele, indicating that both the *Pi2* and *Pi9* genes have not been widely deployed in Chinese *indica* rice cultivars. Since only 57 *japonica* cultivars were screened in the present study, whether the *Pi2* and *Pi9* genes have been widely incorporated into Chinese *japonica* rice cultivars remains to be further investigated. The *indica* restorer line Huazhan, which was identified possessing the *Pi2* allele, was bred from a Malaysian rice cultivar SC02-S6 (China Rice Data Center, http://www.ricedata.cn/variety/varis/607962.htm?607962). Thus, the *Pi2* allele in Huazhan was possibly derived from SC02-S6. In addition to Huazhan, 14 additional accessions displayed a *Pi2*/*Piz-t*-type genotype when screened with the Pi9-Pro marker. Further screening with the Pi2-LRR, Pi2SNP markers revealed that none of the 14 lines possesses the *Pi2* allele, suggesting that these lines possibly carry the *Piz-t* allele. The 14 *Piz-t-*type accessions included four *indica* restorer lines, five *indica* conventional cultivars/breeding materials, and five *japonica* conventional cultivars/breeding materials (Table 2). Thus, the *Piz-t* gene appears to have been incorporated into diverse Chinese rice cultivars. The inoculation experiments showed that the 15 *Pi2*/*Piz-t-*type cultivars were resistant to isolates incompatible to *Pi2* or *Piz-t*. When inoculated with isolates compatible to *Pi2* or *Piz-t*, the *Pi2*/*Piz-t-*type cultivars showed resistance to some isolates. This could be explained by the cultivars possessing other blast *R*-genes conferring resistance to some of the isolates. The inoculation results also showed that compared with the *Piz-t* donor line Toride-1, the *Pi2* donor line C101A51 and the *Pi9* donor line 75-1-127 were resistant to more Fujian isolates (Table 2), suggesting that *Pi2* and *Pi9* have more potential value in improving blast resistance in the Fujian area.

## Conclusion

In this study, we developed two allele-specific markers for *Pi2* and *Pi9*, and evaluated the presence of the two blast *R*-genes in diverse Chinese cultivars/breeding materials based on allele-specific marker analysis. The assessment revealed that *Pi2* and *Pi9* have not been widely incorporated into diverse Chinese *indica* rice cultivars, supporting that the two blast-*R* genes can be novel gene sources for breeding of blast resistant *indica* rice in China. Furthermore, the newly developed markers will be highly useful in tracking *Pi2* and *Pi9* for blast resistance in breeding programs.

## Methods

### Plant materials and blast isolates

Blast *R*-gene donors C101A51 (carrying *Pi2*), Toride-1 (carrying *Piz-t*) and 75-1-127 (carrying *Pi9*) were kindly provided by Dr. Guo-Liang Wang (Department of Plant Pathology, Ohio State University). The 434 rice cultivars and breeding materials (Additional file 2: Table S2) were maintained at the Fujian Provincial Key Laboratory of Genetic Engineering for Agriculture, Fujian Academy of Agricultural Sciences, Fuzhou, China. F_2_ populations derived from crosses between C101A51 and CO39, and between 75-1-127 and CO39 were used to validate the developed markers. The *M. oryzae* isolates PO6-6, KJ201, CHE86, and CHNOS were kindly provided by Dr. Guo-Liang Wang (Department of Plant Pathology, Ohio State University). Seven *M. oryzae* isolates RB16, RB22, HYG99, IR16, JL311, JL501, and ZY838 collected from Fujian province, China, were also used in the study.

### Rice blast inoculations

Rice blast inoculations were performed in a greenhouse with a fogging system to maintain a high level of humidity. Rice seedlings were grown in the greenhouse in cement tanks filled with puddled soil for about 2 weeks under conditions at 20–30 °C with a light and dark cycle of 14 and 10 h, respectively. The *M. oryzae* isolates were grown on oatmeal agar plates in the dark for 7 days at 28 °C and then cultured under light for 5 days at room temperature for sporulation. Before inoculation, the cement tank was partially covered with a plastic membrane tent. Rice seedlings were spray-inoculated with spores at a concentration of 5 × 10^5^ spores mL^−1^. After spraying, the cement tank was completely covered with the tent, and the inoculated seedlings were maintained under dark conditions for 24 h. The tent was then removed from the tank, and the seedlings were grown in the greenhouse under high humidity for a further week for symptom evaluation.

### DNA extraction

Leaf tissues from rice seedlings were collected for DNA extraction. About 100 mg young leaf samples collected in Eppendorf tubes were frozen in liquid nitrogen, and were ground into fine powder with a TissueLyser (Qiagen, Haan, Germany). Rice genomic DNA was extracted from ground tissues by the CTAB (Cetyltrimethyl Ammonium Bromide) method as mainly described by Murray and Thompson (1980).

### DNA sequence analysis

The genomic sequences of the *Pi2/9* locus in the rice cultivars C101A51 (DQ352453), 75-1-127 (DQ285630), and Nipponbare (DQ454158), and the nucleotide sequence of the *Piz-t* gene in the rice cultivar Toride-1 (DQ352040) were retrieved from the GenBank database of the National Center for Biotechnology Information (http://www.ncbi.nlm.nih.gov). Based on a preliminary multiple sequence comparison of DQ352453, DQ285630, DQ454158, and DQ352040, a primer pair P9-F/P9-R (Additional file 1: Table S1) was designed and utilized to amplify the allelic fragments corresponding to the promoter region of *Pi9* and its alleles from C101A51, Toride-1, 75-1-127, Nipponbare, and 22 other rice cultivars (Fig. 1b). Similarly, a primer pair 2-LRR-F/2-LRR-R (Additional file 1: Table S1) was designed to amplify the allelic sequences bearing the polymorphisms for the first six amino-acid differences between Pi2 and Piz-t within the LRR motifs (Zhou et al. 2006). Primers 2-LRR-F/2-LRR-R were subsequently utilized in PCR amplification using genomic DNA templates of C101A51, Toride-1, 75-1-127, Nipponbare and 20 other cultivars (Fig. 2b). PCR products from primers P9-F/P9-R and 2-LRR-F/2-LRR-R were sequenced using an ABI3730 XL automatic DNA sequencer. DNA sequences were aligned using Clustal Omega (Sievers et al. 2011).

### Development of allele-specific markers for *Pi2* and *Pi9*

Based on the multiple sequence alignment of 26 sequences of the promoter region of *Pi9* and its alleles, primers 9-Pro-F/9-Pro-R were designed to develop a marker targeting the InDel polymorphisms among the *Pi2*/*Pizt*, *Pi9*, and non-*Pi2*/*Piz-t*/*Pi9* alleles. PCR was performed under standard conditions with about 50 ng of rice genomic DNA as template. The PCR products were separated in 10 % polylacrylamide gels and then visualized by silver-staining. Based on the multiple sequence alignment of 24 sequences within the LRR coding region of the *Pi2* and non-*Pi2* alleles, a CAPS marker was designed to differentiate the *Pi2* and non-*Pi2* alleles by using the dCAPS Finder 2.0 program (Neff et al. 2002) for the identification of appropriate restriction enzymes. PCR amplicons from primers 2-LRR-F/2-LRR-R were digested with *Pst*I or *Hin*fI. Cleavage of PCR amplicons was performed at 37 °C for 4 h under the following conditions: 10 μL of the PCR amplicon, 2 uL 10 × buffer (TaKaRa, Dalian, China), and 1 uL of the restriction enzymes *Pst*I or *Hin*fI (TaKaRa, Dalian, China) in a final volume of 20 μL. The enzyme-digested products were separated in 10 % polylacrylamide gels and then visualized by silver-staining.

### Genotyping in diverse Chinese rice cultivars or breeding materials with allele-specific markers

Genomic DNAs of the 434 Chinese rice cultivars or breeding materials were subjected to genotyping for the presence of *Pi2* and *Pi9* using the newly developed InDel marker Pi9-Pro and CAPS marker Pi2-LRR. In addition, two previously reported CAPS/dCAPS markers Pi2SNP and Pi9SNP (Hua et al. 2015) which targeted the coding region of *Pi2* and *Pi9* respectively, were applied for genotyping. For genotyping using the Pi2SNP marker, PCR amplicons from primers Pi2SNP-F/Pi2SNP-R (Additional file 1: Table S1) were digested with *Hin*fI. And for genotyping using the Pi9SNP marker, PCR amplicons from primers Pi9SNP-F/Pi9SNP-R (Additional file 1: Table S1) were digested with *Hin*dIII. The enzyme-digested products were separated electrophoretically in 10 % polylacrylamide gels.

## Additional files

Additional file 1: Table S1. Primers used in this study. (DOC 38 kb) Additional file 2: Table S2. Rice cultivars and breeding materials assessed in this study. (DOC 51 kb)

## Abbreviations

CAPS: cleaved amplified polymorphic sequence
CTAB: cetyltrimethyl ammonium bromide
dCAPS: derived cleaved amplified polymorphic sequence
InDel: insertion-deletion
LRR: leucine-rich repeat
MAS: marker-assisted selection
NBS-LRR: nucleotide binding site–leucine-rich repeat
SNP: single nucleotide polymorphism
SSR: simple sequence repeat
