# Supplementary material for: Allele-specific marker-based assessment revealed that the rice blast resistance genes Pi2 and Pi9 have not been widely deployed in Chinese indica rice cultivars
Source: Rice (N Y). 2016 May 4;9:19. doi: 10.1186/s12284-016-0091-8 (PMC4854853; doi:10.1186/s12284-016-0091-8)
Supplement: Additional file 1: Table S1. — Primers used in this study. (DOC 38 kb) [file 12284_2016_91_MOESM1_ESM.doc]

Table S1 List of primers used in this study

| Primers | Sequence: 5ʹ-3ʹ | Expected size (bp) | Comments |
| --- | --- | --- | --- |
| P9-F | CGGTTATAGATGAATATAGTTC | 803 | Forward primer to amplify the fragments corresponding to the promoter Pi9 and its’ alleles region for allele mining |
| P9-R | GTTCGACTCTCCCTTCAAGC | 803 | Forward primer to amplify the fragments corresponding to the promoter Pi9 and its’ alleles region for allele mining |
| 9-Pro-F | TGATTATGTTTTTTATGTGGGG | 111 for *Pi2*/Piz-t allele,  128 for *Pi9* allele,  138 for non-*Pi2/Piz-t*, non-*Pi9* alleles | Forward primer for InDel marker Pi9-Pro |
| 9-Pro-R | ATTAGTGAGATCCATTGTTCC | 111 for *Pi2*/*Piz-t* allele,  128 for *Pi9* allele,  138 for non-*Pi2/Piz-t*, non-*Pi9* alleles | Reverse primer for InDel marker Pi9-Pro |
| 2-LRR-F | CGTTGTATAGGACAGTTTCATT | 436 for *Pi2* allele,  439 for non-*Pi2* alleles | Forward primer for allele mining between *Pi2* and non-*Pi2* alleles, and for CAPS marker Pi2-LRR |
| 2-LRR-R | AATCTAGGCACTCAAGTGTTC | 436 for *Pi2* allele,  439 for non-*Pi2* alleles | Reverse primer for allele mining between *Pi2* and non-*Pi2* alleles, and for CAPS marker Pi2-LRR |
| Pi2SNP-F | TACTCTTCGTTGTATAGGAC | 462 | Forward primer for CAPS marker Pi2SNP |
| Pi2SNP-R | GGAGGAGGAGATGAAATAGAATC | 462 | Reverse primer for CAPS marker Pi2SNP |
| Pi9SNP-F | CGCCGGTTGATAAGTAAAAGCT | 126 | Forward primer for CAPS marker Pi2SNP |
| Pi9SNP-R | CAAGAACTAATATCTACCCATGG | 126 | Reverse primer for CAPS marker Pi2SNP |
