# Supplementary material for: Allele-specific marker-based assessment revealed that the rice blast resistance genes Pi2 and Pi9 have not been widely deployed in Chinese indica rice cultivars
Source: Rice (N Y). 2016 May 4;9:19. doi: 10.1186/s12284-016-0091-8 (PMC4854853; doi:10.1186/s12284-016-0091-8)
Supplement: Additional file 2: Table S2. — Rice cultivars and breeding materials assessed in this study. (DOC 51 kb) [file 12284_2016_91_MOESM2_ESM.doc]

Table S2 Rice cultivars and breeding materials assessed in this study

| **Category** | **Subspecies** | **Cultivar or breeding materials** | **No. of cultivars** |
| --- | --- | --- | --- |
| Donor lines |  | C101A51 (*Pi2*), Toride-1 (*Piz-t*), 75-1-127 (*Pi9*) |  |
| Restorer lines of hybrid rice | *Indica* | 6078, 8L124, 93-11, C162, C418, Ce 64, Changhui 117, Changlizhua, Chenghui 448, Duohui 43, Duoxi No.1, Ehui 928, Enhui 80, Enhui 99-64, Fu 838, Fuhui 016, Fuhui 718, Fuhui 838, Fuhui 964, Gu 154, Guanghui 998, Gui 32, Gui 44, Gui 99, Guixian R1, Hanghui 173, Hang No.1, Huaming 921, Huazhan, Hui 92, Hui 129, Hui 1826, Jianghui 151, Kehui 752, Lehui 188, Luhui17, Mianhui 501-1, Mianyang 436, Minghui 63, Minghui 69, Minghui 72, Minghui 75, Minghui 77, Minghui 81, Minghui 82, Minghui 86, Minghui 88, Minghui 1259, Minghui 2155, Minhui 3301, Minhui 3139, Minhui 3189, Minhui 3229, Minhui 6118, Miyang 46, Nanhui 125, Nanhui 175, Nanhui 397, Nanhui 511, Ninghui 627, P143, R95, R128, R187, R188, R198, R316, R402, R524, R1128, R2070, R3550, R669, Shuhui 215, Shuhui 527, Shuhui 537, Shuhui 881, T230, Wan 3, Wuhui 898, Xianghui 68, Xianhui 207, Xianghui 91269, Yanhui 559, Yuehui 94, Yunhui 72, Zaohui 63, Zaohui 89, Zaohui 3301, Zhenhui 084, Zhonghui 8006 | 91 |
| Maintainer or sterile lines of hybrid rice | *Indica* | 29B, 710S, 58025B, II-32B, Anfeng B, Annongwanjing B, Baoxie-7B, Baoxie 123B, Bobai B, Defeng B, Dixiang B, D62B, D297B, D702B, Erjiunan No.1B, Feng 008B, Fuyi B, G46B, G96 Bao, Gang 46B, Guangzhan 63S, Guangkang 13B, Gufeng B, Hua 1B, Huaxiang B, Hui B, Jin 23B, Jinghu B, Jingu B, Jinnante B, Jinnante 43B, L301B, Liming B, Longtepu B, Luxiang 618B, Mianxiang 1B-61, Minfeng 1B, Peiai 64S, SE21S, Shen 95B, Si B, T55B, Taifeng B, Tianfeng B, V20B, Wufeng B, Xiangai B, Xie B, Yexiang 29B, Yixiang 1B, You 1B, Yue 4B, Yuefeng B, Zaogang B, Zaoshunonghu 6 B, Zhaoyangyihao B, Zhenbai B, Zaote B, Zhong 9B, Zhuzhen B, Zhenshan 97B | 61 |
| Conventional cultivars and breeding materials | *Indica* | 97gk1019, 9311/CBB23, Aihechi, Aijiaonante, Aimazhan, Aimi, Aituogu 151, Aizaizhan, Babaili, Baihehualuo, Baihezaohe, Baimaodao, Baixianghe, Baoxuan No.21, Bawangbian 1, Beizinuo, Bengbanggu, Binwan No.3, Biwusheng, BL123, C84, C418/Fengaizhan, CBB23, Chengduai No.3, Chengnongshuijin, Chihenuo, Chuanxiang 29B/Fengaizhan, CO39, Cunsanli, Dabainuo, Dangyu No.5, Dawannuo, Dazilaishan, Dongtingwanxian, Ergangai, Esiniu, Ezao 11, Fanhaopi, Feidongtangdao, Fengxinzhan, Gaoshan 32, Gaoyangdiandao, Gaoyou 35, Giza 176, gk729, Gongju 73, Guang 265, Guangchao No.1, Guanggangu, Guangluai 15, Guangluai No.4, Guichao No.2, Guisixiangnuo, Hailin No.1, Hanmadao, Haobuka, Haolai, Haoxiang, Hehuadao, Heidao, Heidu 4, Heimi, Hengxianliangchun, Hongainuo, Hongchaxiazhi, Hongganqitou, Hongjiangu, Hongjinghangu, Hongqi No.5, Hongwan No.1, Hongxiangerhao, Hunannuo, Huagujianglai, Huajienuo, Huajingxian 74, Huaiyuantangdao, Huanganzhan, Huanghexi, Huangmaozaohe, Huangnuo 3, Huangsiguizhan, Jiabala, Jiangnongzao No.1, Jiaoshuizao, Jiayu 164, Jiayu 948, Jiazao 935, Jiazao No.1, Jiefangxian, Jiguanzhan, Jinbaoyin, Jinsinuo, Jinxibai, Jinyou No.1, Jinzhinuo, Kajinuo, Kangwenqingzhan, Lamujia, Laohonggu, Laohuzhong, Laolaihuang, Leihuozhan, Liaodong 218, Lijiangxintuanheigu, Liusha No.1, Liushizao, Liuyenian, Lizhishanlan No.1, Longpingzhili, Lucaihao, Lunhui 422, Luoqianjin, Luyu No.132, M3122/IR50, Magunuo, Mamagu, Maweinian, Meihuanuo, Menggudao, Menjiading No.2, Menjiagao No.1, Menjiaheisi, Minbeiwanxian, Momi, Mowanggu, Muguanuo, Muxiqiu, Naergu, Nanbagu, Nanjing No.11, Nantehao, Nanxiongzaoyou, Niumunuo, Nongxiang 16, Peiai 64, PeiC122, Poya, Putaohuang, Qihegu, Qingke, Qinglongweiliao, Qingsiai 16B, | 225 |

**Supplementary Table 1. (Continued from previous page)**

| **Category** | **Subspecies** | **Cultivar or breeding materials** | **No. of cultivars** |
| --- | --- | --- | --- |
| Conventional cultivars and breeding materials | *Indica* | Qiuqianbai, Qishirigu, Qitoubaigu, Qitougu, Ruanmigu, Sanbailaishan, Sanbaili, Sankecun, Shendao9810, Shennong 265, Shufeng 101, Shuyazhan, Simiao, Suyunuo, Taiguosimiao, Taiyou, Taizhongxianxuan 2, Taizhongzailai 1, Teqing, Teqing No.2, TQ347, Sibeiti-Balilla, Tumangdao, Wanlixian, Wannuo, Wuhezhan, Wujuhonggu, Wuhongxian, Wunong No.1, Wuyuehong, Xiangaizao 10, Xiangbai 99, Xiangdao, Xiangwanxian No.1, Xiangwanxian No.7, Xiaobaigu, Xiaohuangma, Xiaojiugu, Xiaoyanggu, Xibainian, Xidaohuang, Xikangxiaobaigu, Xinyouzaosheng, Xuanerchangtan, Xuebaoxiangnuo, Xugunuo, Yangdao No.2, Yanghenuo, Yanshuichi, Yeweipan, Yinchaozhan, Yigendao, Yingbobainandu, Yixianhong, Yizhixiang, Youzhan, Yueyezhan No.6, Yuxian No.7, Yuxian No.8, Yuyannuo, Zaoshuiyinzhan, Zaoxian1061, Zegu, Zhe 733, Zhefu 802, Zhenxian 232, Zhong 413, Zhongjian 100, Zhonglouyihao, Zhongnong No.4, Zhou 903, Zihui 100/IKAN468, Zimangfeie, Zinuo |  |
| Conventional cultivars and breeding materials | *Japonica* | 02428, Baimangjing, Baizhujing, Dandongludao, Funingzipi, Guanghexiangnuo, Guanghuahuang, Haobayong No.1, Haomake (K), Heimangdao, Heijing No.2, Heizhujing, Hongguzi, Huanghezaoer, Huhui 628, Jindao 1, Jing 7623, Katy, Laoguangtou 83, Laohongdao, Lemont, Lianjing No.7, Liaojing 287, Lixinjing, Longhuamaohu, M3122/Dianjing, Maguzi, Mojing, Nantiangangjiu, Nianhenuo, Ninghui 21, Nipponbare, Shanjiugu, Shuiyuan 290, Shuiyuan 300 li, Shuiyuan 377, Sujing No.2, Taibei 309, Taichung No.65, Taidongludao, Tetepu, Tieganwu, Weiguo, Wuzidui, Xiangjing 9707, Xiangnuo, Xingguo, Xiushui 13, Xiushui 115, Yelicanghua, Youmangzaojing, Yunyin, Zhengdao No.5, Zhonghua No.8, Zhonghua No.13, Zhonghua No.15, Zhonghua No.16 | 57 |
| **Total** |  |  | **434** |
